# Book Reviews

**Published:** 1832-08

**Authors:** 


### BIBLIOGRAPHICAL NOTICE.

*An Essay on the Epidemic Cholera; being an Inquiry into its new or Contagious Character; including Remarks on the Treatment, as likewise Tables of the average Rate of Disease and of Mortality recently occurring in London.* By JOHN WEBSTER, M.D. &c.—12mo. pp. 220. Burgess and Hill, London.

THE original parts of this volume are well written, and the others cleverly compiled. On the whole, it is an ingenious publication, in which all that has been advanced, and all that can be said, in defence of the non-communicability of the " blue cholera" has been collected and condensed, and reduced to a convenient and readable bulk. We recommend its perusal to the partisans (if there be any) of both sides of the question: to the non-contagionists, as the best breviary extant, by which they may judge of the strength or weakness of their position; and to the contagionists, that they may perceive how much can be said, and how little can be proved, against the doctrines they defend.

If any thing had been wanting to settle our belief in the communicability of cholera, Dr. WEBSTER's book, by shewing how little all the combined arguments of the non-contagionists really amount to, would have greatly assisted in confirming our creed: for, notwithstanding the author's elaborate declarations of his utter impartiality, he continually prejudges the question; and notwithstanding his anxiety to persuade the reader of his unswerving, unbiassed course, he raises chimeras which he mistakes for antagonists, and claims a victory when he has only vanquished a visionary foe. For example, can the following be fairly said to be essentially characteristic of any, even the most *contagious*, diseases? can such be considered an unbiassed statement of the tenets of any of the advocates of the contagious nature either of cholera or of any other communicable disease?

" If this disease was actually of such a nature as to be communicated by one individual to another, the question naturally presents itself, where, and by what means was cholera originally produced? as it must, at some period or other, have had a beginning; and if any cause, or any series of influences, could at one time originate the malady, the same might at another, with equal facility: since what acted upon one human being, and induced diseased action, might, with equal reason, do so to a thousand, without the aid of personal communication. Besides, if the disease was really of that contagious nature some have, in the warmth of their zeal, believed; should this pestilence once begin in a country, it could never have an end, so long as there remained a single man alive to be affected; under which awful circumstances there must be an end to population, since the disease ought thus constantly to increase; but the reverse is often the fact, for it has been observed, when appearing most extensively, the cholera has suddenly diminished." (P. 101.)

We know that some wild visionaries deny the contagious powers of every disease, even of variola and lues: but we do not believe Dr. Webster to be among the number; for he admits the communicability of typhus, which many physicians doubt. Now can it be seriously contended that neither psora nor syphilis are communicable diseases, *because* they must have had an origin, and the first person who ever suffered from them could not have caught them from an antecedent sufferer? Can it be seriously said that smallpox is not communicable, because persons are frequently exposed to its influence, and yet escape from an attack? we know of many such instances, and a medical friend was witness to a still more remarkable occurrence; for of ten (if not more) persons unprotected either by vaccination or previous attacks of smallpox, who were exposed to its malign influence, one only had the disease, and all the rest remained exempt.

Of what *contagious nature some have believed this disease to be*, we know not, if the Doctor has correctly stated their opinions, when he says of the cholera in question, "should this pestilence once begin in a country, it could never have an end, *so long as there remained a single man alive to be affected*; under which awful circumstances here must be an end to population, since the disease ought thus constantly to increase." Such arguments can never require a specific confutation; they carry their own answer with them. Do neither variola, nor scarlatina, nor yellow fever, nor plague, ever cease *while a single man remains alive to be affected?*

We had marked many other passages for comment, in which a similar, we doubt not unintentional, distortion of the doctrines of those who believe cholera to be communicable, is made; but we think it will be scarcely necessary to extract them; for if such be the author's idea of the essential signs of contagion and characteristics of a contagious disease, we will confess ourselves at once to be non-contagionists; for we know not of any disorder, whether cholera or not, which shews them. But if by a contagious disease be meant a disease which, whether, like typhus, it frequently originates spontaneously, and is subsequently communicable and communicated from man to man; or whether, like syphilis, variola, and psora, its usual mode of propagation be from individual to individual, although doubtlessly it must have had at first some other source; then indeed do we find in cholera, such as has raged in India, such as has ravaged Persia and Russia, such as has afflicted this country and France, such as appears now to be depopulating Canada, just those very signs which characterize the diseases we have named, and which diseases we contend to be contagious.

Let us suppose for sake of example (that our meaning may not be misunderstood,) that two persons suffering with symptoms of malignant cholera were taken to one of the public receptacles in this town; that the usual attendants were absent or engaged, and that another person in good health, say the matron, in the absence of the nurse, officiated; put the patients to bed, gave the medicines, rubbed in the embrocations, and so forth; and that this matron, who had not been near the sources where the sick had contracted the disease, nor been subjected to any other suspected causes, nevertheless shortly afterwards became affected with cholera in its most virulent form, and died; we say, supposing such a case to occur, would it not appear probable, and more than merely probable, that the patients had communicated the disease to the matron? This, it behoves us to declare is not merely an imaginary case, but one of very recent date; one, indeed, which has only just occurred. (Vide p. 155.)

We rather think the learned Doctor does really consider some such cases as these to be what he calls "triumphant proofs," although, to our minds, it is not a subject that admits of triumph, whichever opinion may be shewn to be correct; and he must, indeed, have been conscious of the weakness of his cause when he stopped the press to add, in an appendix, what he is pleased to call the "Declaration of the Westminster Medical Society;" for they who were present on the 28th of April, know that this declaration was not brought forward until after the usual hour of adjournment had passed; that no notice had been given of the intention to move any resolution; that most of the members had quitted the room; and that when, after a very strong opposition from nearly half of the few that were left, and in spite of the *protest* of the president, it was forced upon the meeting, it was only carried by a majority of four, and at a time of night when the theatre, which had frequently been crowded to excess, was almost empty, and when but forty members were present: and yet this majority of four, this twenty-two against eighteen, is set forth as the "*opinion of the majority of the Society,*" the decision of twenty-two put forth as the decision of the majority of a society, consisting of between eight and nine hundred members, upwards of eight hundred of whom could know nothing of what twenty-two were doing for them, until the public prints informed them that they had decided on a question that had never been submitted to their decision.

We remember somewhere to have read of a certain judge, who never wished to hear arguments on two sides of any question; for he declared candidly, that, although he could make up his mind after the speech of the first advocate, he was always puzzled when he heard the second counsel: so likewise here, they who *wish* to be non-contagionists will do well to peruse Dr. Webster's book, and it alone; and they who wish to arrive at the truth, will do well to read it also; but to these latter we would speak in a warning voice, "*audite alteram partem.*"

---

*Practical Observations on the Medicinal Virtues of the Beulah Saline Spa, Norwood; and on the Disorders in which it is most efficacious, especially those connected with Derangements of the Digestive Organs.* By Archibald Maxfield, Member of the Royal College of Surgeons in London, and formerly House-Surgeon to St. Bartholomew's Hospital.—8vo. pp. 82. Highley.

The Beulah water has been analysed by Messrs. Faraday and Hume, and one quart of it is found to contain the following proportions of ingredients:

| | |
|---|---|
| Sulphate of magnesia . . . . | 123 grains |
| Sulphate of soda and magnesia . | 32 |
| Muriate of soda . . . . . . . | 19 |
| Muriate of magnesia . . . . | 18½ |
| Carbonate of lime . . . . . | 15 |
| Carbonate of soda . . . . . | 3 |

Mr. Maxfield states that, when first drawn from the well, the water is inodorous, pellucid, and sparkling; it possesses a saline and slightly bitter flavour: it contains a quantity of carbonic acid gas, which makes a valuable addition to its powers as a medicine; its temperature is 52° Fahr., and its specific gravity 1011. It may be administered with two intentions: first, either to empty the bowels simply, or to produce a merely laxative effect; or, secondly, to promote an increased secretion from the internal surface of the intestines, or, in other words, to induce a purgative operation. It is also, according to Mr. Maxfield, a "diuretic and febrifuge." He has made a careful comparison of this water with all the other mineral springs hitherto discovered, that have attained any degree of celebrity, and he has no hesitation in stating that the Beulah spa is the most potent and valuable specimen of a saline spring this country possesses, and is deserving higher reputation than any of them as a medicine. While he estimates thus highly the medical virtues of this spring, the author confesses, with proper impartiality that we must not overlook its collateral advantages, in the beauty of the situation, the salubrity of the air, the benefit derived from exercise, novelty of scene, and agreeable occupation.

The natural beauties of Norwood, and the surrounding country, are indeed highly attractive, and admirably adapted to restore serenity to the ruffled and exhausted mind, and health and vigour to an enfeebled body. By our cockney travellers these attractions are not properly appreciated, and many of them, at much unnecessary expense and inconvenience, roam to a great distance for those advantages which are presented close to the metropolis in the delightful vicinity of Norwood.

